# Anticholinesterase Activity and Bioactive Compound Profiling of Six Hop (*Humulus lupulus* L.) Varieties

**DOI:** 10.3390/foods13244155

**Published:** 2024-12-22

**Authors:** Bartłomiej Sagan, Bogusław Czerny, Anna Stasiłowicz-Krzemień, Piotr Szulc, Urszula Skomra, Tomasz M. Karpiński, Jolanta Lisiecka, Adam Kamiński, Aleksandra Kryszak, Oskar Zimak-Krótkopad, Judyta Cielecka-Piontek

**Affiliations:** 1 Department of Neurosurgery and Pediatric Neurosurgery, Pomeranian Medical University Hospital No. 1 in Szczecin, Unii Lubelskiej 1, 71-252 Szczecin, Poland; bartlomiejsagan@gmail.com; 2 Department of General Pharmacology and Pharmacoeconomics, Pomeranian Medical University in Szczecin, Żołnierska 48, 70-204 Szczecin, Poland; 3 Department of Pharmacognosy and Biomaterials, Poznan University of Medical Sciences, Rokietnicka 3, 60-806 Poznan, Poland; astasilowicz@ump.edu.pl (A.S.-K.); jpiontek@ump.edu.pl (J.C.-P.); 4 Department of Agronomy, Poznań University of Life Sciences, Dojazd 11, 60-632 Poznań, Poland; piotr.szulc@up.poznan.pl; 5 Institute of Soil Science and Plant Cultivation State Research Institute, Department of Biotechnology and Plant Breeding, Czartoryskich 8 Str., 24-100 Puławy, Poland; urszula.skomra@iung.pulawy.pl; 6 Department of Medical Microbiology, Poznań University of Medical Sciences, Rokietnicka 10, 60-806 Poznań, Poland; tkarpin@ump.edu.pl; 7 Department of Vegetable Crops, Faculty of Agronomy, Horticulture and Bioengineering, Poznan University of Life Sciences, Dabrowskiego 159, 60-594 Poznan, Poland; jolanta.lisiecka@up.poznan.pl; 8 Department of Orthopedics and Traumatology, Pomeranian Medical University Hospital No. 1, Pomeranian Medical University in Szczecin, Unii Lubelskiej 1, 71-252 Szczecin, Poland; adam.kaminski@pum.edu.pl; 9 Department of Pharmacology and Phytochemistry, Institute of Natural Fibres and Medicinal Plants, Wojska Polskiego 71b, 60-630 Poznan, Poland; aleksandra.kryszak@iwnirz.pl (A.K.); oskar.zimak-krotkopad@iwnirz.pl (O.Z.-K.)

**Keywords:** hop, neuroprotection, acetylcholinesterase, butyrylcholinesterase, xanthohumol

## Abstract

Hops (*Humulus lupulus* L.) are widely recognized for their use in brewing, but they also possess significant pharmacological properties due to their rich bioactive compounds, with many varieties exhibiting diverse characteristics. This study investigates the chemical composition and biological activities of extracts from six hop varieties, focusing on quantifying xanthohumol and lupulone using High-Performance Liquid Chromatography (HPLC) and Total Phenolic Content (TPC) analysis. The hop varieties demonstrated significant variability in bioactive compound concentrations, with Aurora showing the highest xanthohumol (0.665 mg/g) and Zwiegniowski the highest lupulone (9.228 mg/g). TPC analysis revealed Aurora also had the highest phenolic content (22.47 mg GAE/g). Antioxidant activities were evaluated using DPPH, ABTS, CUPRAC, and FRAP assays, with Aurora and Oregon Fuggle displaying the most potent capacities. Aurora, in particular, showed the highest activity across multiple assays, including significant acetylcholinesterase (AChE), butyrylcholinesterase (BChE), and tyrosinase inhibition, with IC_50_ values of 24.39 mg/mL, 20.38 mg/mL, and 9.37 mg/mL, respectively. The chelating activity was also assessed, with Apolon demonstrating the strongest metal ion binding capacity (IC_50_ = 1.04 mg/mL). Additionally, Aurora exhibited the most effective hyaluronidase inhibition (IC_50_ = 10.27 mg/mL), highlighting its potential for anti-inflammatory applications. The results underscore the influence of genetic and environmental factors on the bioactive compound profiles of hop varieties and their biological activity offering promising avenues for pharmaceutical and nutraceutical applications. However, further studies are needed to fully understand the potential interactions between hop cones components.

## 1. Introduction

Hops, scientifically known as *Humulus lupulus* L., stand as a ubiquitous raw material found abundantly across the globe. This versatile plant, belonging to the *Cannabaceae* family, extends its reach far and wide, playing a pivotal role in various cultural and industrial landscapes. As a crucial ingredient in brewing, the hop’s widespread presence not only imparts its distinctive bitterness, flavors, and aromas to beers but also intricately weaves a rich tapestry of tradition and craftsmanship that spans continents [1]. Besides their role in brewing, hops are recognized for their mild sedative properties and are present in herbal remedies, dietary supplements, and in over-the-counter drugs for anxiety, tension, and difficulty sleeping [2]. Hops contain a wide variety of biologically active substances: bitter acids, essential oils, and polyphenols [3]. In hops, the production of resins and oils primarily occurs in the lupulin glands, which are small, yellowish glands found in the cone-like structures mainly present in hop flower [4,5]. These lupulin glands are concentrated mainly in the bracts and scales of the hop cone [4].

Bitter acids, also called soft resins, are chemically diprenylated (alpha acids) like humulone, adhumulone, cohumulone, or triprenylated (beta acids) derivatives, for example, lupulone, colupulone, and adlupulone of phloroglucinol and its homologues [6]. They owe their name to their bitter taste, which aids digestion, but this is not their only feature. Research has confirmed that alpha acids (humulones) are responsible for the calming properties of hops [3]. These compounds are also particularly effective against Gram-positive bacteria [7]. Compared to phenol, the activity of alpha acids is about 200 times stronger, and beta acids are 800 times stronger. This intense effect is related to the hydrophobic nature of these molecules, which favors interactions with microbial cell membranes. In the second half of the 20th century, lupulone was used to treat tuberculosis [8]. Additionally, soft resins also have antioxidant, anti-inflammatory, and anticancer properties [9]. They have the ability to induce apoptosis, inhibit induced tumor promotion in vivo, as well as angiogenesis, i.e., the formation of capillaries [3]. Beta acids are more effective in inhibiting the growth of cancer lesions and reducing proliferation than alpha acids [3]. Hop polyphenols were divided into, flavonols (quercetin, kaempferol, and myricetin), flavan-3-ols ((+)-catechin, (−)-epicatechin, and (+)-gallocatechin), phenolic carboxylic acids (ferulic acid, caffeic acid, vanillic acid, gallic acid, protocatechuic acid, sinapic acid, 4-hydroxybenzoic acid, p-coumaric acid), and other phenolic compounds (prenylflavonoids (xanthohumol, isoxanthohumol, desmethylxantohumol, and 6- and 8-prenylnaringenin, stilbenoids (resveratrol), and so on). These compounds represent a substantial category of biologically active secondary metabolites, constituting approximately 3% to 6% of the dry weight of hop cones [10]. The primary reservoirs of these polyphenols are the strobiles and bracts, except for prenylflavonoids, which are released from lupulin glands alongside bitter acids and essential oils [11]. Prenylflavonoids, a class of flavonoid compounds characterized by the presence of a prenyl group, are distributed among various plant species. The majority of natural prenylflavonoids exhibiting antioxidant activity are sourced from the *Moraceae*, *Fabaceae, Apiaceae*, *Asteraceae*, *Cannabaceae*, and *Euphorbiaceae* plant families [12]. Notably, hops stand out as a prominent source of prenylflavonoids, with xanthohumol being a specific example. It is an antioxidant with activity significantly more potent than vitamin C, exhibiting a strong antimicrobial potential, along with substantial anticancer potential and additional anti-inflammatory and neuroprotective effects [13]. Hop cones also contain essential oils that are responsible for many of the fragrant components of hops, along with α- and β-acids that serve as precursors to bittering agents [14]. Hop essential oils are volatile, non-polar fractions containing unique compounds, including terpenoids, alkanes, alcohols, and esters [15]. Studies have identified key aroma compounds in hop cones, such as myrcene, humulene, caryophyllene, linalool, and 3-methylbutanoic acid, which play significant roles in the aroma profile of different hop varieties [16]. Additionally, nonanal, methyl nonanoate, and 3-methylbutyl 2-methylpropanoate also play a role as prominent odorants found in the fresh, dried hop cones, and pelletized hops [17]. Hop essential oils exhibit significant antimicrobial, antioxidant, and anti-inflammatory activities, making them effective against a variety of pathogens, free radicals, and inflammatory conditions [18,19,20]. Hop essential oils also demonstrate sedative, anticancer, and antispasmodic properties [2,21,22,23]. Different plant varieties exhibit distinct secondary metabolite compositions, which directly impacts their biological activity [24,25,26,27,28]. These metabolites are influenced by genetic factors, environmental conditions, and developmental stages [29]. Variations in metabolite levels can alter a plant’s medicinal properties, flavor, or resistance to pesticides and diseases [30,31,32].

This paper aimed to study five cultivars of *H. lupulus*—Galena, Zwiegniowski, Cerera, Aurora, Apolon, and Oregon Fuggle. The hop cones were analyzed for their total phenolic content, as well as their xanthohumol and lupulone levels. Additionally, the antioxidant and chelating potential of the cultivars was investigated, alongside their ability to inhibit acetylcholinesterase, butyrylcholinesterase, tyrosinase, and hyaluronidase. Furthermore, their antimicrobial potential was evaluated. This study focused on in vitro neuroprotective properties. While the in vitro findings are promising, further validation through in vivo studies and exploration of the underlying mechanisms, such as in cell line studies, are essential to confirm the neuroprotective potential of these cultivars.

## 2. Materials and Methods

### 2.1. Chemical Reagents

Lupulone and xanthohumol were sourced from Sigma-Aldrich, Poznan, Poland. Trifluoroacetic acid and HPLC-grade methanol were provided by Merck, Darmstadt, Germany. High-quality purified water was produced using the Direct-Q 3 UV system from Millipore (Molsheim, France; model Exil SA 67120). 5,5-dithio-bis-(2-nitrobenzoic acid), 2,2-Diphenyl-1-picrylhydrazyl (DPPH), ferric chloride hexahydrate, 2,2′-azino-bis(3-ethylbenzothiazoline-6-sulfonic acid), neocuproine, 2,4,6-Tri(2-pyridyl)-s-triazine, trolox, acetylcholinesterase from *Electrophorus electricus* (AChE), butyrylcholinesterase from equine serum (BChE), azelaic acid, acetylcholine iodide (ATCI), butyrylcholine iodide (BTCI), Trizma^®^ hydrochloride and Trizma^®^ base, bovine serum, hexadecyltrimethylammonium bromide, hyaluronic acid, L-DOPA, and tyrosinase from mushrooms were obtained from Sigma-Aldrich, Schnelldorf, Germany. The 0.1 M phosphate buffer at pH 6.8 was supplied by STAMAR. Sodium chloride was purchased from Avantor Performance Materials, Gliwice, Poland. Ammonium acetate and methanol were acquired from Chempur, Piekary Śląskie, Poland. Cupric chloride dihydrate, 99.5% acetic acid, 96% ethanol, and sodium acetate trihydrate were obtained from POCH, Gliwice, Poland.

### 2.2. Plants Materials

Six hop cultivars of different origin and belonging to the aroma or bitter type were used in the study (Table 1). Plants were grown under natural field condition in the Polish hop germplasm collection maintained in the Agricultural Experimental Station of the Institute of Soil Science and Plant Cultivation in Puławy (Poland) (51°24′19″ N, 21°57′32″ E, altitude 116 m) on medium-heavy alluvial soil. The average 30-year period (1991–2020) temperature for this location is 8.9 °C, and the average annual rainfall is 592 mm. The crop year 2021 was characterized by a temperature slightly below average (8.6 °C), while the amount of rainfall was 30% higher than average (768 mm). Plants were grown at a spacing 1.5 × 3.0 m on a supporting structure 7 m high. Soil cultivation, fertilization, and plant protection against pest and diseases were carried out in accordance with the principles of integrated pest management [33]. Hop cones were collected at the time of technological maturity BBCH 89 (from the end of August to the beginning of September 2021) from the upper part of the plants (from a height of about 5 m). For each cultivar, the cones from three randomly selected plants were taken, and then cones were mixed, and a bulk sample was prepared. The collected material was air-dried at room temperature.

### 2.3. Extraction Process

Each variety of hop cones was grounded and extracted with 70% (*v*/*v*) methanol in a 1:20 ratio (*m*/*v*). The extraction was carried out for 30 min at a temperature of 40 °C using an ultrasonic bath (constant, uninterrupted sonication, frequency 37 kHz, ultrasonic peak max. 800 W) (Thermo Fisher Scientific, Waltham, MA, USA). The resulting extract was filtered. The extraction process was repeated two more times (each time using fresh extractant was used). The extracts were subsequently concentrated using a rotary vacuum evaporator under reduced pressure at 40 °C, evaporating the solvent until each extract reached a final concentration of 150 mg/mL.

### 2.4. Chromatographic Analysis

The levels of xanthohumol and lupulone were quantified using high-performance liquid chromatography coupled with a diode array detector (HPLC-DAD) (Shimadzu Corp., Kyoto, Japan). The method, previously described and validated by the authors, was employed for the analysis [34]. For the determination, a ReproShell PFP column (150 mm × 4.6 mm; 2.7 µm) as the stationary phase was used. The mobile phase consisted of 0.1% trifluoroacetic acid (solvent A) and methanol (solvent B). The elution gradient was set up as follows: 0–5 min B: 50–60%, 5–15 min B: 60–70%, 15–20 min B: 70%, 20–25 min B: 70–80%, 25–30 min B: 80–85%, and 30–35 min B: 85%. The flow rate was maintained at 1.0 mL/min, and the column temperature was set at 40 °C. The injection volume was 10.0 µL, with detection carried out at a wavelength of 323 nm. The retention time for xanthohumol was approximately 11.7 min, while lupulone was detected at around 17.6 min. Chromatographic data were acquired and processed using LabSolutions LC software (version 1.86 SP2, Shimadzu Corp., Kyoto, Japan).

### 2.5. Analysis of Total Phenolic Content

The total phenolic content was analyzed using a modified Folin–Ciocalteu method, which was previously described [35]. Plant extracts or gallic acid solutions were mixed with Folin–Ciocalteu reagent and sodium carbonate, incubated, and the absorbance at 760 nm was measured to express the results as gallic acid equivalents (GAE) per gram of plant material.

### 2.6. Antioxidant Activity

Antioxidant defenses are vital for mitigating oxidative stress, an important contributor to neurodegenerative processes [36]. By neutralizing reactive oxygen species, which, when accumulated, lead to neuronal damage, inflammation, and dysfunction, antioxidants play a pivotal role in preserving neuronal integrity and function. The antioxidant activity of the extracts was evaluated using four distinct assays: 2,2-Diphenyl-1-picrylhydrazyl (DPPH), 2,2′-Azino-bis(3-ethylbenzthiazoline-6-sulfonic acid) (ABTS), cupric reducing antioxidant capacity (CUPRAC), and ferric reducing antioxidant power (FRAP). Prior to each assay, the extracts’ antioxidant activity was screened by testing them at decreasing concentrations. Trolox was used as a reference standard, with its antioxidant activity measured across appropriate concentration ranges for each assay. A linear regression equation was derived from the Trolox concentration, and its corresponding scavenging percentage or absorbance, depending on the specific assay. The antioxidant activity of the extracts in all four assays was reported as milligrams of Trolox equivalent per gram of plant material. [37,38,39].

The DPPH assay procedure was already described [40]. It involved mixing plant extract or Trolox with DPPH solution, incubating in the dark, and measuring absorbance at 517 nm to calculate the DPPH radical inhibition percentage. The ABTS assay was another method used to evaluate the samples’ radical scavenging potential [41]. The assay, conducted as previously described, involved mixing the extract or Trolox with ABTS•+ solution, incubating in the dark, and measuring absorbance at 734 nm to calculate the ABTS radical inhibition percentage. The CUPRAC and FRAP assays were employed to assess the reducing potential of the extracts, following protocols previously published [42]. These assays, conducted as previously described, involved measuring the color change due to reduction of the neocuproine–copper (II) complex at 450 nm and the reduction of Fe^3+^ to Fe^2+^ with TPTZ at 593 nm, respectively, to assess the reducing potential of the extracts.

### 2.7. Chelating Activity

The Fe^2+^ chelation ability of the extracts was assessed using a method adapted from Studzińska-Sroka et al. [43].

The Fe^2+^ chelating activity was determined by incubating the extract with iron (II) chloride and ferrozine, measuring absorbance at 562 nm, and calculating the chelation percentage using the appropriate formula:

(1) $${Fe}^{2+}\;chelating\;activity\left(\%\right)=1-\frac{A_{s}-A_{bs}}{A_{c}-A_{bc}}\times 100$$

where

*A_s_* is the absorbance of the sample, *A_bs_* is the absorbance of the blank of the sample, *A_c_* is the absorbance of the control, and *A_bc_* is the absorbance of the blank of the control. From the obtained results, the IC_50_ value was calculated.

### 2.8. Anticholinesterase Activity

The potential of the extracts to inhibit enzymes linked to neurodegeneration, such as AChE and BChE, was investigated. AChE and BChE are enzymes involved in the breakdown of acetylcholine, a neurotransmitter crucial for memory and cognitive function [44]. Inhibiting these enzymes can increase acetylcholine levels, which may improve cognitive function and potentially protect against neurodegenerative diseases like Alzheimer’s. Since the dysfunction of acetylcholine transmission is a hallmark of such conditions, the ability of extracts to inhibit AChE and BChE suggests their potential neuroprotective effects, possibly aiding in the management or prevention of neurodegeneration. Initially, the inhibitory activity of the extracts was screened by measuring their effect at descending concentrations. Strong inhibitors of esterases include rivastigmine, donepezil, and galantamine [45], while potent tyrosinase inhibitors are hydroquinone, kojic acid, and azelaic acid [46]. Consequently, galantamine was selected as the standard inhibitor for AChE and BChE, and azelaic acid was chosen for tyrosinase inhibition.

The inhibition of AChE and BChE was determined following the method described in previous studies [47]. The inhibition of AChE and BChE was assessed by measuring the color change of thiocholine, released during enzymatic reactions with artificial substrates—acetylthiocholine iodide (ATCI) for AChE and butyrylthiocholine iodide (BTCI) for BChE—coupled with 5,5′-dithio-bis-(2-nitrobenzoic) acid (DTNB), which forms the 3-carboxy-4-nitrothiolate anion (TNB). The percentage inhibition was calculated based on the absorbance at 405 nm:

(2) $$\mathrm{AChE}/\mathrm{BChE}\;\mathrm{inhibition}\;(\%)=\frac{1-(A_{1}-A_{1b})}{\left(A_{0}-A_{0b}\right)}\times 100\%$$

where

*A*_1_—the absorbance of the test sample;

*A*_1*b*_—the absorbance of the blank of the test sample; 

*A*_0_—the absorbance of control;

*A*_0*b*_—the absorbance of the blank of control.

### 2.9. Antityrosinase Activity

Tyrosinase is involved in neurodegenerative diseases such as Parkinson’s disease by oxidizing excess dopamine, leading to the formation of dopamine quinones, which are reactive molecules that cause neuronal damage and cell death [48]. Inhibiting tyrosinase could, therefore, be a potential strategy for preventing or treating these diseases. The tyrosinase inhibition was assessed using a previously established method [34]. The assay evaluates the reduction in color intensity caused by the inhibition of enzyme activity, where L-DOPA is used as a substrate, and the inhibition percentage and IC_50_ value are determined based on absorbance measurements at 475 nm. The level of tyrosinase inhibition was calculated using the equation:

(3) $$\mathrm{Tyrosinase}\;\mathrm{inhibition}\;(\%)=\frac{1-(A_{1}-A_{1b})}{\left(A_{0}-A_{0b}\right)}\times 100\%$$

where

*A*_1_—the absorbance of the test sample;

*A*_1*b*_—the absorbance of the blank of test sample;

*A*_0_—the absorbance of control;

*A*_0*b*_—the absorbance of the blank of control.

### 2.10. Inhibition of Hyaluronidase

The hyaluronidase inhibition was assessed using a modified version of the turbidimetric method described by Grabowska [49]. The exact protocol used in this study was published earlier [40]. The inhibition of hyaluronidase was calculated using the following equation:

(4) $$I\%=\frac{\left(P-B_{3})-(B_{2}-B_{1}\right)}{\left(B_{4}-B_{3}\right)-(B_{2}-B_{1})}\times 100\%$$

The turbidity of the sample (*P*) was measured, with the following blanks used for comparison: *B*_1_ (absorbance of the blank control), *B*_2_ (absorbance with enzyme and hyaluronic acid for enzyme property assessment), *B*_3_ (absorbance with enzyme and test extract), and *B*_4_ (absorbance with hyaluronic acid and test extract).

### 2.11. Antimicrobial Activity

In the study, clinical strains of *Staphylococcus aureus*, *Pseudomonas aeruginosa*, and *Candida albicans* were used. The bacterial strains were cultured on tryptic soy agar (TSA), and yeast on Sabouraud agar (Graso Biotech, Starogard Gdański, Poland), at 36 °C for 24 h. The minimal inhibitory concentrations (MICs) of the extracts were determined using the microdilution method in 96-well plates (Nest Scientific Biotechnology, Wuxi, China). The studies were conducted following the methodology described in our previous publications in tryptic soy broth (TSB) (Graso Biotech, Starogard Gdański, Poland) [50,51]. Serial dilutions of hop flower extracts were prepared, starting from a concentration of 150 mg/mL. Additionally, as a control substance, the antiseptic octenidine dihydrochloride (Schülke & Mayr, Norderstedt, Germany) was used, starting from a concentration of 50 µg/mL.

### 2.12. Analysis of the Results

For the statistical analysis, Statistica 13.3 software (StatSoft Poland, Krakow, Poland) was used. Data are expressed as means ± standard deviations. The normality of each distribution was assessed using skewness and kurtosis tests, while Levene’s test was used to evaluate the equality of variances. One-way analysis of variance (ANOVA) was performed, followed by the Bonferroni post hoc test to compare the experimental outcomes for each extract. Statistical significance was set at *p* < 0.05. Principal component analysis (PCA), conducted using PQStat v.1.8.4.140 software (Poznań, Poland), was employed to explore the relationships between compound profiles and their biological activity. The Pearson correlation matrix was also calculated using PQStat v.1.8.4.140 software.

To identify the extract with the greatest neuroprotective potential, which encompasses antioxidant activity (measured by DPPH, ABTS, CUPRAC, FRAP methods), chelating properties and the inhibition of AChE, BChE, and hyaluronidase enzymes, a multidimensional comparative analysis (MCA) was performed. This analysis compares multiple features and organizes results based on synthetic indicators. Destimulants were converted into stimulants during this process. The diagnostic features were normalized and standardized for evaluation, and synthetic measures were calculated to rank the regions accordingly.

## 3. Results and Discussion

The experimental work commenced with the extraction of six hop varieties, which were subsequently characterized using HPLC and TPC analysis. The HPLC analysis specifically focused on quantifying the levels of xanthohumol and lupulone, two compounds known for their biological activities. The concentrations of these compounds varied significantly among the different hop varieties, potentially influencing their biological activities (Table 2). For Xanthohumol, Aurora exhibited the highest concentration at 0.665 mg/g, followed by Cerera and Apolon with 0.656 mg/g and 0.654 mg/g, respectively. In terms of Lupulon, the Zwiegniowski variety had the highest content at 9.228 mg/g, significantly higher than the other varieties. This variability in compound levels could have implications for the use of these hop extracts in various applications, such as pharmaceuticals and nutraceuticals.

Among the six hop varieties analyzed, Aurora exhibited the highest total phenolic content, measuring 22.47 ± 1.15 mg GAE/g (Table 3). This indicates that Aurora possesses a significant concentration of polyphenolic compounds, which are known for their antioxidant properties and potential health benefits. Additionally, Cerera showed notable total phenolic content at 20.26 ± 0.88 mg GAE/g.

Literature studies examining various *H. lupulus* varieties have revealed notable differences in TPC levels. For example, Jae Il Lyu et al. explored the impact of extraction methods on TPC in hop cultivars, noting that ethanol extraction generally resulted in higher TPC levels (ranging from 57.00 to 81.90 mg GAE/g dry weight) compared to water extraction methods (ranging from 52.80 to 64.50 mg GAE/g dry weight) [52]. Moreover, Kobus-Cisowska et al. provided further insights into TPC variability across specific hop varieties such as Magnum, Marynka, and Lubelski, revealing significant differences in TPC values (ranging from 3083.9 to 4666.9 µg/g dry weight) [53]. These variations highlight the influence of genetic and environmental factors on phenolic compound production in hops. Additionally, Clara María Albani et al. found that hop leaves of Mapuche and Victoria varieties exhibited higher TPC values (74 ± 4 µg GA/mL and 62 ± 3 µg GA/mL extract, respectively) compared to Bullion, Cascade, and Traful varieties [54]. Extracts were prepared with 50% methanol, from 1 g of leaves submerged in 30 mL of solvent.

Another step of the study involved evaluating antioxidant activity to assess the potential. This was achieved using various assays, such as DPPH, ABTS, CUPRAC, and FRAP (Table 4)

The antioxidant activity results revealed that the Oregon Fuggle variety exhibited the highest DPPH scavenging activity at 59.94 ± 0.40 mg Trolox/g, followed closely by Aurora with 57.66 ± 0.57 mg Trolox/g, indicating strong free radical neutralization. Aurora demonstrated the greatest antioxidant capacity in the ABTS and CUPRAC assays with values of 41.36 ± 1.08 mg Trolox/g and 66.44 ± 0.73 mg Trolox/g, respectively. Aurora also showed the greatest activity in the FRAP assay with 44.34 ± 0.88 mg Trolox/g. Overall, Aurora and Oregon Fuggle varieties stood out for their superior antioxidant activities across multiple assays. There is also data about different hop varieties’ antioxidant activity in the literature. In a study by Bilska et al., a water extraction process at 70 °C was used to obtain extracts from the hop cones of the Magnum and Lubelski cultivars. The DPPH radical scavenging activity was found to be greater in the extract from the Magnum cones (4.21 ± 0.09 mg TE/g dry weight) compared to the extract from the Lubelski cones (3.87 ± 0.05 mg TE/g dry weight) [55]. The Herkules cultivar of hop cones from Germany’s Hallertau region was used to prepare the concentrated hop extract using ethanol (50:50 *v*/*v*) and ultrasound, demonstrating significant ferric reducing antioxidant power (1284 µmol Fe^2+^Eq g^−1^ dm) and Trolox equivalent antioxidant capacity (757 µmol g^−1^ dm) [56]. In another study, the antioxidant activities of various extracts obtained from *H. lupulus* L. cones collected from Balıkesir were evaluated using DPPH, ABTS, FRAP, and CUPRAC assays [57]. The n-hexane extract showed the strongest DPPH scavenging activity (14.95 ± 0.03 μg Trolox equivalent/g sample). The ethanol extract exhibited the highest CUPRAC activity (3.15 ± 0.44 mmol Trolox equivalent/g sample). Methanol-2 and methanol-3 extracts demonstrated potent ABTS (7.35 ± 0.03 mM Trolox equivalent) and FRAP (1.56 ± 0.35 mmol Fe^2+^/g sample) activities, respectively. In another study, among hop varieties tested for antioxidant activity using DPPH and ABTS assays, the results also showed significant variations [53]. The Magnum variety again exhibited the highest antioxidant activity in both DPPH and ABTS assays among the varieties studied (MW: DPPH 4.75 mmol Tx/g dw, ABTS 1.32 mmol Tx/g dw), followed by Marynka and Lubelski varieties in the water extracts. In the ethanol extracts, Magnum (ME: DPPH 4.12 mmol Tx/g dw, ABTS 2.33 mmol Tx/g dw) still showed high antioxidant activity, followed closely by Marynka and Lubelski varieties. The choice of a solvent for extracting hop cones is crucial, as it influences the profile of compounds present in the extract [19,58]. In a study by Lyu et al., the El Dorado variety showed higher antioxidant activity in ethanol extracts (DPPH: IC_50_ 124.3 µg/mL; ABTS: IC_50_ 95.4 µg/mL) compared to other varieties (Calypso, Cascade, Cluster, Magnum, Saaz1, Saaz2, Saaz3, Saaz4) [52]. The correlation was between DPPH of ethanol extract and its total flavonoid compound and its total phenolic compound. Moreover, the correlation was between ABTS of ethanol extract and its total flavonoid compound and its total phenolic compounds. No significant correlation was observed between antioxidant activities, total phenolic compound, and total flavonoid compound in water extracts.

The chelating activity involves the binding of metal ions, preventing them from catalyzing harmful oxidative reactions, whereas antioxidant stress refers to the neutralization of free radicals to reduce cellular damage, offering complementary protective mechanisms against oxidative stress.

**Figure 1:**
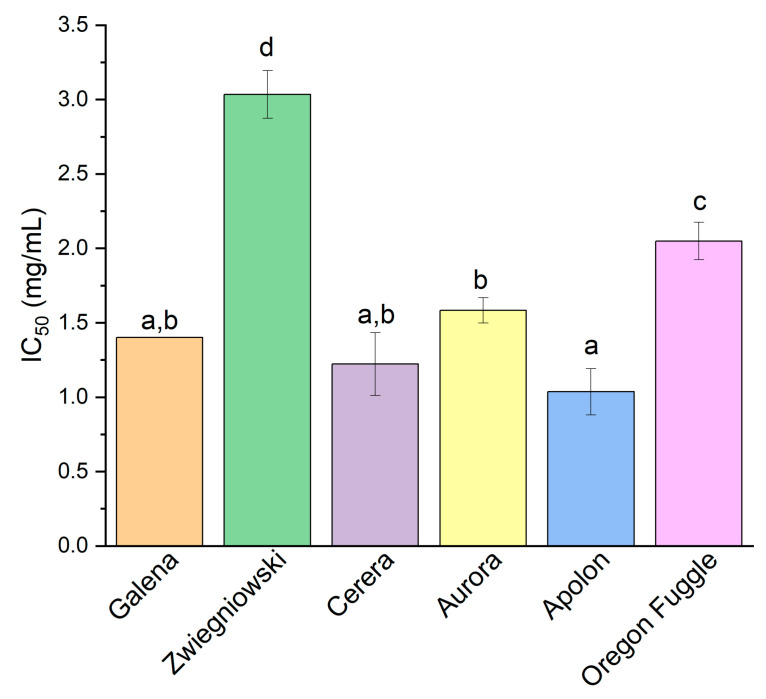
The chelating activity of hop flower extracts of different varieties is presented as IC_50_ (mg/mL). Different letters (a–d) within the bars indicate statistical differences (*p* < 0.05).

Apolon exhibited strong chelating activity with an IC_50_ value of 1.04 ± 0.16 mg plant material/mL, indicating effective metal ion binding (Figure 1). Aurora showed slightly lower but still significant chelation with an IC_50_ value of 1.58 ± 0.09 mg plant material/mL. Oregon Fuggle demonstrated moderate a chelating activity with an IC_50_ value of 2.05 ± 0.13 mg plant material/mL, suggesting its capability to bind metal ions, though with less potency compared to Apolon and Aurora. The results obtained for Galena, Cerera, and Apolon cultivars were better than for a reference substance, quercetin, whose IC_50_ is 1.46 mg/mL (calculated as prepared concentration) [43]. Polyphenols are capable of binding transition metal ions by forming complexes through their electron–donor groups [59]. The process initiates with the oxidation of the polyphenol, which leads to the removal of a hydrogen atom and the generation of a phenoxyl radical. This radical then interacts with the metal ion to create a stable complex [60]. Literature confirms the chelating properties of hop cones. In a study of dried hop cones from Magnum, Lubelski, and Marynka cultivars, extracts were prepared using water and ethanol–water solutions [53]. Ethanol extracts from Magnum showed the highest iron ion chelation activity (55.43–88.76%), with activity increasing with extract concentration. This chelating ability was significantly related to the levels of ferulic acid, epicatechin, syringic acid, and p-coumaric acid. In a Kowalczyk et al. study, chelating activity was assessed for hop extracts from Magnum and Marynka cultivars using various solvents, with hydroalcoholic extracts (50% ethanol or methanol) generally showing higher metal chelating activity compared to aqueous extracts [61]. Among the hop products, type-45 (T45) pellets of Magnum cultivar demonstrated superior chelating power, achieving the lowest EC_50_ values and indicating higher metal ion binding efficiency. The chelating ability of hop extracts was significantly higher for those obtained using supercritical CO_2_ extraction at elevated temperatures and pressures, whereas ethanol extracts from the remaining plant material exhibited notably poorer results compared to the scCO_2_ extracts [34].

In the anticholinesterase activity study, the Aurora variety exhibited the most potent inhibition against AChE with an IC_50_ value of 24.39 ± 1.27 mg plant material/mL (Table 5). Compared to the reference substance galantamine, the ability of hop varieties to inhibit the enzyme was weaker. For BChE inhibition, the Aurora variety again demonstrated significant potency with an IC_50_ of 20.38 ± 0.61 mg plant material/mL, but the result was worse than for the reference.

The literature confirms the concentration-dependent cholinesterase inhibition by hop cone extracts, Kobus-Cisowska showed that from the Magnum, Lubelski, and Marynka cultivars, with both water and ethanol extracts showing higher activity against acetylcholinesterase (AChE) than butyrylcholinesterase (BChE), particularly in water extracts [53]. Another study assessed the anticholinesterase activity of hop essential oil and its main components, including α-humulene, β-myrcene, and β-caryophyllene, finding that β-caryophyllene was the only substance with significant activity, exhibiting a dose-dependent inhibition with an IC_50_ value of approximately 18.05 mg/mL [62]. In Nascimento et al., the acetylcholinesterase inhibition tests revealed that only hexane extracts of hop flowers, particularly from the Herkules variety, and the standard international calibration extract of hop ICE-4 (standard mixture of bitter acids, purity 69.2%) acted as effective inhibitors, with halo sizes of 6 to 9 mm, indicating significant anticholinesterase activity, while methanol extracts, obtained using a Soxhlet system, did not show comparable inhibition [63]. The concentration dependence of anticholinesterase activity and the variability in effectiveness among extracts obtained with different solvents were also confirmed by Paventi et al. [64]. Hop cones from Bojano, Italy, were dried, ground, and extracted with methanol, acetone, or n-hexane. The hop extracts showed dose-dependent inhibitory activity on AChE with IC_50_ values of 0.331 ± 0.025 μg/mL for n-hexane, 0.440 ± 0.108 μg/mL for methanol, and 0.505 ± 0.041 μg/mL for acetone extracts. In hop cone extracts prepared using supercritical carbon dioxide, the most significant anticholinergic effects were observed with those prepared at 50 °C and 5000 PSI [34]. These extracts demonstrated IC_50_ values of 9.759 ± 0.433 mg/mL for acetylcholinesterase (AChE) and 2.661 ± 0.021 mg/mL for butyrylcholinesterase (BChE). In contrast, ethanol extracts prepared from the same material after supercritical CO_2_ extraction showed fewer effective results.

**Figure 2:**
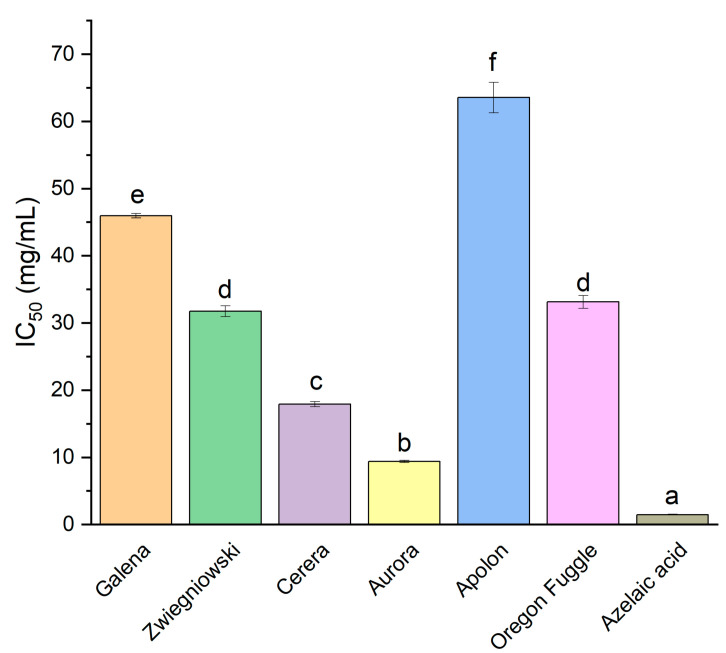
Inhibition of tyrosinase, by hop flower extracts of different varieties presented as IC_50_ (mg/mL). Different letters (a–f) within the bars differ significantly (*p* < 0.05).

For tyrosinase inhibition, Aurora exhibited the next strongest activity with an IC_50_ value of 9.37 mg/mL (Figure 2). Cerera showed moderate inhibition with an IC_50_ value of 17.9 mg/mL. Azelaic acid, with an IC_50_ value of 1.47 mg/mL, served as the reference compound, which was the most potent inhibitor of tyrosinase activity. The literature confirms the capability of *H. lupulus* constituents to inhibit tyrosinase. Methanol extract from *H. lupulus* demonstrated inhibition of mushroom tyrosinase, while seven isolated flavonoids exhibited potent suppression of monophenolase (IC_50_ range: 15.4–58.4 µM) and diphenolase (IC50 range: 27.1–117.4 µM) functions of the enzyme [65]. Kinetic analyses using Lineweaver–Burk and Dixon plots identified chalcones as competitive inhibitors, whereas flavanones acted through mixed and non-competitive mechanisms. Another investigation reported that hop tannins inhibited tyrosinase with an IC_50_ of 76.52 ± 6.56 μM, demonstrating a competitive–uncompetitive mixed mode of inhibition based on kinetic evaluations [66]. Similarly, extracts from the aerial parts of *H. japonicus* also displayed tyrosinase inhibitory effects [67].

**Figure 3:**
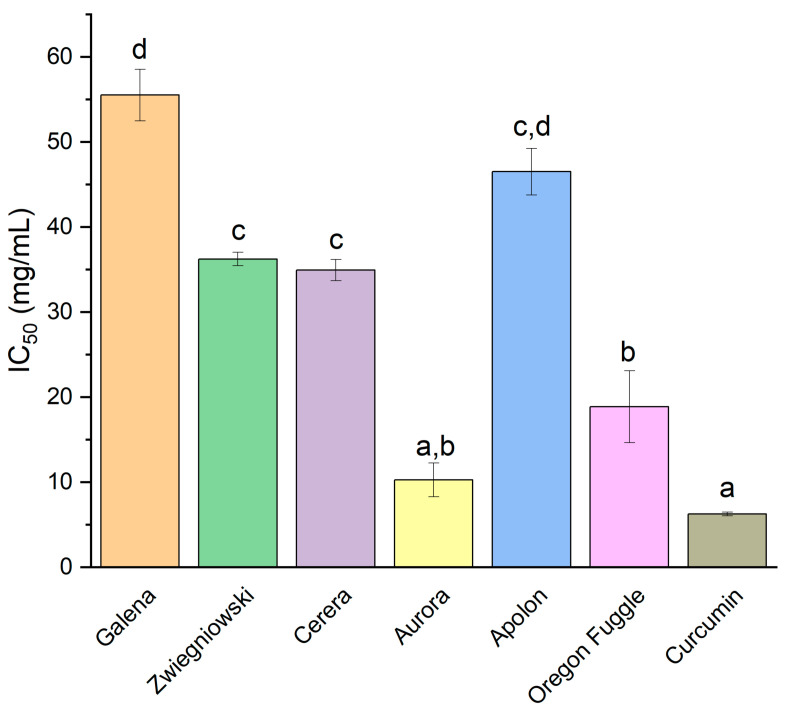
Inhibition of hyaluronidase, by hop flower extracts of different varieties presented as IC_50_ (mg/mL). Different letters (a–d) within the bars differ significantly (*p* < 0.05).

For hyaluronidase inhibition, Aurora exhibited the most potent activity with an IC_50_ value of 10.27 ± 3.99 mg/mL, suggesting its robust capability to inhibit the enzyme responsible for hyaluronic acid degradation (Figure 3). All cultivars were worse than curcumin (IC_50_ = 6.25 mg/mL) at inhibiting hyaluronidase. However, the result obtained for the Aurora cultivar was statistically similar. This property highlights Aurora’s potential to reduce inflammation by preserving hyaluronic acid levels in tissues. Recent research has also highlighted the promising anti-inflammatory properties of hop extracts and their components. In Liu et al., hop flowers were extracted using ethyl acetate to isolate compounds with hyaluronidase inhibitory activity [68]. The isolated compounds, including rutin (IC_50_ = 4.2 µM), quercetin (IC_50_ = 8.5 µM), kaempferol (IC_50_ = 6.7 µM), and isorhamnetin (IC_50_ = 5.4 µM), were further purified and analyzed through NMR spectroscopy. Among these, rutin exhibited the strongest inhibition of hyaluronidase. Rho iso-alpha acids, a modified hop extract known for its anti-inflammatory properties, selectively inhibited inducible COX-2 with an IC_50_ value of 1.3 µg/mL, showing over 200-fold selectivity compared to COX-1, which has an IC_50_ greater than 289 µg/mL [69]. The hop extract from *H. lupulus*, rich in α-bitter acids (47.8%), β-bitter acids (23.6%), and essential oils (7.8%), showed significant anti-inflammatory activity in vitro [70]. At 4 µg/mL, it reduced p-ERK phosphorylation similarly to hydrocortisone at 20 µg/mL, though hydrocortisone had a stronger effect on p-p38 phosphorylation (*p* ≤ 0.05). The extract also decreased IL-6 and IL-8 secretion in irradiated primary human keratinocytes, with hydrocortisone showing a more pronounced reduction in IL-8 (*p* ≤ 0.05). Supercritical CO_2_ hop extract significantly reduced IL-6 production, an inflammatory cytokine, in UV-irradiated human primary keratinocytes (HPKs), demonstrating strong anti-inflammatory effects with an IC_50_ of 0.8 µg/mL [71]. In another study, the hydroalcoholic extract of Cascade hops demonstrated the ability to inhibit the release of IL-8, a pro-inflammatory cytokine, in a concentration-dependent manner, with a notably lower IC_50_ value compared to the aqueous extract [72]. This inhibition was linked to the reduction in NF-κB-driven transcription, a key regulator of inflammatory responses, suggesting that the anti-inflammatory effect of the hops extract is mediated through the suppression of NF-κB activity. There are also studies focused on xanthohunol anti-inflammatory activity. Xanthohumol demonstrated anti-inflammatory activity by significantly reducing interleukin-6 expression in compressively stimulated cementoblasts [73]. It also modulated inflammatory signaling pathways by decreasing the phosphorylation of extracellular signal-regulated kinase and protein kinase B.

The tested hop flower extracts exhibited weak antimicrobial activity compared to the antiseptic octenidine dihydrochloride (Table 6). They were most effective against the yeast *Candida albicans*, with the most frequent MIC levels at 75 mg/mL. The extracts showed weaker activity against the Gram-positive *Staphylococcus aureus*, with MIC values of 150 mg/mL, and the weakest effect against the Gram-negative *Pseudomonas aeruginosa.* Antimicrobial activity and neuroprotective potential in plant extracts may, but do not have to, correlate, as they involve distinct molecular mechanisms and biological targets; thus, a plant extract may contain bioactive compounds with either or both properties, but their efficacy in one domain does not necessarily predict effectiveness in the other, and different fractions of the same plant extract may exhibit divergent antimicrobial and neuroprotective activities due to the selective extraction of distinct bioactive compounds [74,75].

**Figure 4:**
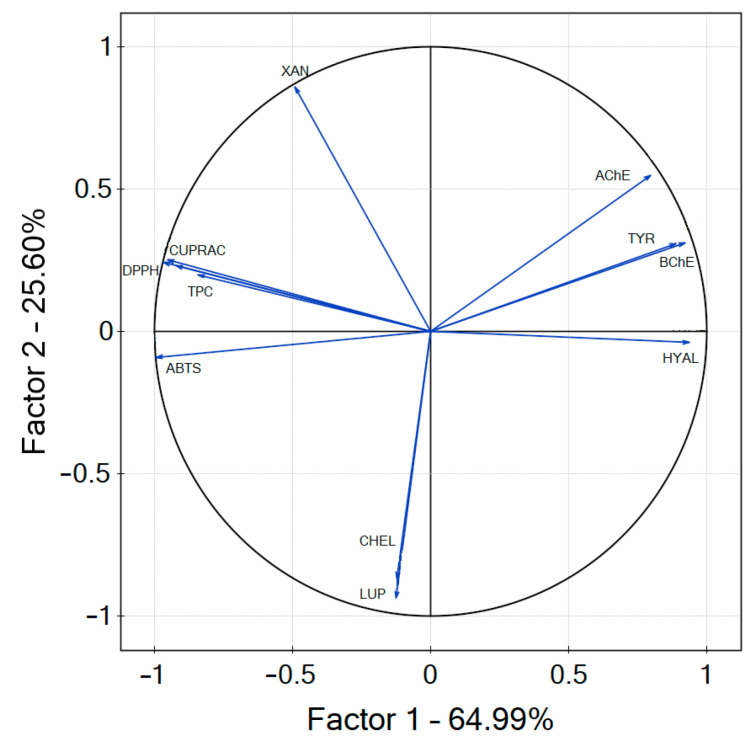
Contributions of variables—xanthohumol (XAN), lupulone (LUP) content, Total Phenolic content (TPC), ABTS, DPPH, FRAP, CUPRAC, chelating (CHEL), acetylcholinesterase (AChE), butyrylcholinesterase (BChE), hyaluronidase (HYAL), tyrosinase (TYR) to PCs.

PCA was conducted to examine the relationships between the plant material, the levels of secondary metabolites, and the biological activity of the extracts. Factor 1 (PC1) accounted for 64.03% of the total variation in the samples, while factor 2 (PC2) explained 26.96% of the variation (Figure 4). Inhibition of AChE, BChE, and hyaluronidase are the parameters that correlate positively with PC1; all of the other parameters correlate negatively with PC1. DPPH, FRAP, CUPRAC, TPC, the content of xanthohumol, and inhibition of the enzymes correlate positively with PC2, while ABTS, chelating potential, and the content of lupulone correlate negatively with PC2. The antioxidant activity is strongly positively correlated to TPC (correlation coefficient > 0.7) and had a good positive correlation with xanthohumol content, as well as being strongly negatively correlated to BChE and inhibition of hyaluronidase as they were presented as IC_50_ values [76]. Multidimensional comparative analysis is an effective method for evaluating and comparing extracts based on a variety of properties. This approach takes into account several factors, including the inhibition of enzymes such as AChE, BChE, tyrosinase and hyaluronidase, chelating potential, and antioxidant activities measured through ABTS, DPPH, FRAP, and CUPRAC assays. By examining these aspects, the extract demonstrating the highest neuroprotective potential was identified, with enzyme inhibition being a key factor in combating neurodegeneration. In the overall ranking, the Aurora cultivar was identified as having the greatest neuroprotective potential.

*Lupuli flos* is widely used in the brewing industry for its bittering and preservative properties and also in dietary supplements. It is also present in pharmaceutical preparations as a pharmacopoeial herbal drug [77]. Safety for herbal medicinal products is assessed based on longstanding traditional use (30 years globally, including at least 15 years in the EU) or well-established use with at least 10 years of documented medicinal use and supporting bibliographic safety data, as required by the Herbal Medicinal Products (HMPC) guidance [78]. The HPMC recognizes *Lupuli flos* as traditionally used for relieving mental stress and aiding sleep [79]. In combination with *Valerianae radix*, it is classified under well-established use for a “relief of sleep disorders” and traditional use for alleviating mild mental stress and supporting sleep [80]. The literature confirms that compounds found in *Lupuli flos* can penetrate the blood–brain barrier, which is essential for neuroprotective activity. For instance, 6-prenylnaringenin successfully crossed the BBB in mice [81]. In vivo studies also confirm that xanthohumol crosses the BBB [82]. Based on in vitro studies on parallel artificial membrane permeability assay (PAMPA) model, both xantohumol and lupulone have the ability to cross BBB [34]. *Lupuli flos* extract and its constituents demonstrate a neuroprotective potential, highlighting the preventative promise of hop-derived compounds for neurodegenerative and cognitive disorders, as shown in the literature. For instance, xanthohumol at doses of 0.2 and 0.4 mg/kg mitigated focal brain ischemia and improved neurobehavioral deficits in rats subjected to middle cerebral artery occlusion [83]. Xanthohumol reduced infarct volume, suppressed TNF-α, iNOS, hypoxia-inducible factor, and caspase-3 expression, and inhibited platelet aggregation, thereby demonstrating anti-inflammatory and anti-apoptotic effects [83]. It also activates the Nrf2-ARE signaling pathway, promoting phase II enzyme expression and protects cells against oxidative damage from hydrogen peroxide and 6-hydroxydopamine [84]. It also reduces excitotoxicity caused by glutamate and kainic acid, preserving mitochondrial function through enhanced Mfn-2 and Bcl-2 expression [85]. In aged mice, xanthohumol reduced pro-inflammatory and pro-apoptotic markers, improved synaptic marker expression, and enhanced cognitive functions [82,86]. It also decreased amyloid-β accumulation and tau hyperphosphorylation in Alzheimer’s model, alleviating endoplasmic reticulum stress and oxidative stress [87]. Iso-α-acids improved memory and cognition in mice with hippocampal inflammation, reducing cytokines like IL-1β and TNF-α while inhibiting NF-κB signaling [88,89,90]. In human trials, mature hop bitter acids improved mood, reduced mental fatigue, and enhanced verbal memory recall [91]. The studies conducted in this article are in vitro, offering valuable insights into the bioactive compound profiles and biological activities of various hop varieties. The findings are promising and suggest that hops, particularly the Aurora variety, possess significant antioxidant, enzyme inhibitory properties. However, to further substantiate their potential for pharmaceutical applications, future research is needed. This includes studies in cell lines and in vivo models to explore the biological mechanisms behind the observed effects, assess bioavailability, confirm efficacy in reducing neurodegenerative biomarkers, and determine safety profiles for clinical use. Additionally, investigating possible synergistic effects with other bioactive compounds could enhance the therapeutic potential of hops.

## 4. Conclusions

Hop cones from various cultivars offer diverse bioactive profiles, making them valuable for developing nutraceuticals with tailored health benefits. The study reveals significant variability in the bioactive compound content and biological activities across different hop varieties. While other varieties, such as Oregon Fuggle, also exhibited notable antioxidant properties, Aurora’s superior antioxidant capacity and effective enzyme inhibition make it particularly advantageous for pharmaceutical and nutraceutical applications, including neuroprotection. Various hop extracts showed differing antibacterial activities against specific microbial strains. These findings emphasize the importance of selecting hop varieties based on their specific bioactive profiles to optimize their use in nutraceuticals and pharmaceutical applications. For the latter, future studies should focus on cell line experiments to further explore the biological mechanisms, as well as in vivo models to confirm the bioavailability and efficacy of hop cone bioactives. Additionally, research should include toxicological and pharmacokinetic evaluations to ensure safety and optimize dosage. 

## Figures and Tables

**Table 1:** The hop cultivars used in the study.

Cultivar	Assignment	Origin
Apolon	bitter	Slovenia
Aurora	aroma	Slovenia
Cerera	aroma	Slovenia
Galena	bitter	USA
Oregon Fuggle	aroma	USA
Zwienigowski	aroma	Russia

**Table 2:** The content of xanthohumol and lupulone of hop flower extracts in different varieties, presented as mg of the compound/g plant material. Different letters (^a–e^) within the same column indicate statistical differences (*p* < 0.05).

Variety	Xanthohumol	Lupulone
	Amount of Active Compound (mg)/Dry Plant Material (g)	
Galena	0.554 ± 0.008 ^b^	4.124 ± 0.025 ^c^
Zwiegniowski	0.494 ± 0.006 ^a^	9.228 ± 0.073 ^e^
Cerera	0.656 ± 0.010 ^c^	6.248 ± 0.029 ^d^
Aurora	0.665 ± 0.009 ^c^	3.182 ± 0.026 ^b^
Apolon	0.654 ± 0.014 ^c^	0.702 ± 0.006 ^a^
Oregon Fuggle	0.651 ± 0.008 ^c^	3.969 ± 0.028 ^c^

**Table 3:** Total phenolic content in hop flower extracts prepared from different varieties (mg GAE/g). Different letters (^a–d^) within the same column indicate statistical differences (*p* < 0.05).

Variety	Total Phenolic Content
	mg GAE/g
Galena	9.92 ± 0.38 ^a^
Zwiegniowski	10.99 ± 0.49 ^a,b^
Cerera	20.26 ± 0.88 ^d^
Aurora	22.47 ± 1.15 ^d^
Apolon	12.91 ± 0.41 ^b^
Oregon Fuggle	17.24 ± 0.67 ^c^

**Table 4:** The antioxidant potential of hop flower extracts in different varieties, presented as mg trolox/g plant material, was studied in the DPPH, ABTS, CUPRAC, and FRAP assay. Different letters (^a–e^) within the same column indicate statistical differences (*p* < 0.05).

Variety	DPPH	ABTS	CUPRAC	FRAP
	mg Trolox/g Plant Material			
Galena	28.49 ± 0.30 ^a^	27.02 ± 0.62 ^a^	33.48 ± 0.55 ^a^	22.79 ± 0.25 ^a^
Zwiegniowski	35.46 ± 0.26 ^b^	31.37 ± 0.64 ^b^	38.53 ± 0.62 ^b^	26.33 ± 1.20 ^b^
Cerera	45.62 ± 0.22 ^c^	37.14 ± 0.96 ^c^	52.09 ± 0.62 ^c^	35.76 ± 0.18 ^c^
Aurora	57.66 ± 0.57 ^d^	41.36 ± 1.08 ^d^	66.44 ± 0.73 ^e^	44.34 ± 0.88 ^e^
Apolon	35.19 ± 0.62 ^b^	24.48 ± 0.45 ^a^	38.83 ± 0.42 ^b^	26.20 ± 0.46 ^b^
Oregon Fuggle	59.94 ± 0.40 ^e^	36.63 ± 0.48 ^c^	63.04 ± 0.54 ^d^	39.66 ± 0.49 ^d^

**Table 5:** Inhibition of acetylcholinesterase (AChE) and butyrylcholinesterase (BChE) by hop flower extracts of different varieties presented as IC_50_ (mg/mL). Different letters (^a–f^) within the same column differ significantly (*p* < 0.05).

Variety	AChE	BChE
	IC _ 50 _ (mg/mL)	
Galena	79.50 ± 0.69 ^e^	36.84 ± 2.07 ^d^
Zwiegniowski	46.56 ± 0.56 ^c^	42.62 ± 1.62 ^e^
Cerera	46.56 ± 0.97 ^c^	29.40 ± 0.28 ^c^
Aurora	24.39 ± 1.27 ^b^	20.38 ± 0.61 ^b^
Apolon	143.82 ± 4.59 ^f^	62.09 ± 3.36 ^f^
Oregon Fuggle	56.02 ± 1.99 ^d^	25.03 ± 1.17 ^c^
Galantamine	0.024 ± 0.001 ^a^	0.163 ± 0.004 ^a^

**Table 6:** Antibacterial activity of hop flower extracts of different varieties presented as minimal inhibitory concentration (MIC; mg/mL).

Extract or Compound	MIC [mg/mL]		
	*Staphylococcus aureus*	*Pseudomonas aeruginosa*	*Candida albicans*
Galena	150	150/>150	75
Zwiegniowski	150	150/>150	75
Cerera	150	150	75
Aurora	150	150/>150	75
Apolon	150	150/>150	75/150
Oregon	150	150/>150	75
Octenidine dihydrochloride	0.4–1.6 µg/mL	0.4–3.1 µg/mL	0.2–1.6 µg/mL

## Data Availability

Data are available in a publicly accessible repository.
